# Employee Retention and Change Management During Times of Uncertainty

**DOI:** 10.3389/fpsyg.2022.916709

**Published:** 2022-05-30

**Authors:** Muhamad Ekhsan, Achmad Sudiro, Mugiono Mugiono, Ananda Sabil Hussein

**Affiliations:** Management Department, Faculty of Economics and Business, Universitas Brawijaya, Malang, Indonesia

**Keywords:** employee retention, change management, COVID-19, talent management, employer branding, employee engagement, organizational trust

## Introduction

Human resource management is a type of management that focuses on maximizing the abilities of its employees or members through a number of strategic steps in order to improve employee performance and help the company achieve its goals. According to Mathis et al. (2015). Human resource management has a decisive role in the life of an organization, namely how well the organization performs, how well the organization's strategy can be implemented, and how far the predetermined goals can be achieved. There is a good way to tell how well a company is able to deal with problems if there is a company. Research and discussion on organizational behavior (OB) have been around for about half a century. However, as OB points out, in reality, things never really change, which clearly shows the problems facing managers in an organization have been around since the dawn of civilization (Luthans, 2012). Although the problems in an organization and the solutions provided have not changed much, the emphasis and context of the surrounding environment have certainly changed. For example, from the 1980's to the mid-1990's, managers were busy restructuring their organizations to increase productivity and meet competitive challenges in international markets and increase customer buying interest. A case in point, an analysis of Fortune 500 companies between 1995 and 2005 found the most prominent initiatives were restructuring (downsizing), cost reduction programs, creating shared services, and Six Sigma quality programs (Luthans, 2012).

Based on the literature review, both theoretically and empirically (can be seen in Table 1), this study comes up with hypotheses that will lead to an empirical research model. In his explanation, Wayne (2014) explains that there is a significant influence between employer branding, management performance, and employee retention. This finding is supported by research conducted by Sutherland et al. (2002) that employer branding has a direct influence on employee retention. Even in research conducted by Chhabra and Sharma (2014), employer branding is one of the corporate strategies in modern times that is used to reduce costs from company employee search. Oladapo (2014) explains that talent management has a positive and significant influence on employee retention. This is supported by research conducted by Deery (2008), which states that talent management and work-life balance have a strong enough influence on employee retention. The research conducted by Fahlevi et al. (2020) is limited to companies engaged in hospitality. However, there are previous studies that have obtained quite the same results regarding the relationship between talent management and employee retention in companies as the research conducted by Hughes and Rog (2008).

**Table 1:** Path analysis.

**Path**	**Original sample (O)**	**Sample mean (M)**	**Standard deviation (STDEV)**	**T statistics (|O/STDEV|)**	***P*-Values**
Employee engagement -> employee retention	0.301	0.302	0.137	2.201	0.028
Employer branding -> employee engagement	0.489	0.508	0.117	4.175	0.000
Employer branding -> employee retention	0.133	0.123	0.169	0.788	0.431
Employer branding -> organizational trust	0.230	0.232	0.135	1.709	0.088
Organizational trust -> employee retention	0.494	0.503	0.158	3.131	0.002
Talent management -> employee engagement	0.271	0.259	0.125	2.162	0.031
Talent management -> employee retention	−0.003	0.000	0.200	0.013	0.990
Talent management -> organizational trust	0.619	0.615	0.145	4.277	0.000
Employer branding -> employee engagement -> employee retention	0.147	0.152	0.075	1.955	0.043
Talent management -> organizational trust -> employee retention	0.306	0.322	0.159	1.928	0.045
Talent management -> employee engagement -> employee retention	0.082	0.080	0.058	1.402	0.162
Employer branding -> organizational trust -> employee retention	0.114	0.106	0.062	1.826	0.049

Figurska and Matuska (2013) found that there is a positive and significant influence between employer branding and employee engagement. Their research reveals that employer branding is one of the company's strategies for maintaining their human resources in order to improve the performance of individuals in a company so that its reputation is enhanced. from the company will make the employee bond better. Kennedy and Daim (2010) explain that there is a significant effect between employee engagement and employee retention. In their research, it is explained that employee engagement can be a major force in maintaining employee resilience and causing a decrease in turnover, but this research is limited to companies that operate in the IT industry. Kashyap and Rangnekar (2016) found that there is a positive and significant relationship between employer branding and organizational trust. It is explained that the perception of a company's reputation has a significant influence in reducing employees' willingness to switch companies. I can mediate between the two relationships. Organizational trust is seen as the output of good employer branding in a company.

Al-Hussaini et al. (2019) explain that there is a significant influence between talent management and employee engagement. This also applies to individual performance variables. This study specifically explains how companies develop talent management to increase bonds between employees, which will have an impact on increasing individual performance. This study also explains the role of talent management as a strategy and the role of employee engagement as a mediator between the two relationships. Özçelik (2015) explains the determinants of organizational trust in talent management in an organization. In this case, talent management can also be interpreted as talent management regarding how the company's efforts to maintain fuel retention of its employees will reduce recruitment costs. Research conducted using nurses in a hospital found that there is a direct influence between organizational trust and employee retention. Researchers not only used organizational trust variables but also added job satisfaction variables in measuring the effect on employee retention.

Suikkanen (2010) explained that employee branding can have a direct and indirect effect on employee retention. The role of employee engagement in mediating the effect of these two variables has a significant and positive influence, so employee engagement is needed to increase employee retention. The same thing was also found by Özçelik (2015) in his research. Backhaus and Tikoo (2004) explain the role of organizational trust in mediating the relationship between employer branding and employee retention, but the research conducted by Backhaus and Tikoo (2004) is only conceptual, and there are no empirical findings that explain the role of organizational trust as a mediator. Research conducted by Biswas and Suar (2016) also explains the factors and impacts of employer branding on organizational trust and employee retention. In support of this hypothesis, no in-depth research has been conducted on the role of organizational trust in mediating the effect of employer branding on employee retention.

Bhatnagar (2007) explains that employee engagement can mediate the influence of employer branding on employee retention. The explanation used by the researcher is that talent supported by work ties will have a greater influence on employee retention. This is in line with research conducted by Hughes and Rog (2008), although the research is limited to companies in the hotel industry. Chitsaz-Isfahani and Boustani (2014) explain that organizational trust can mediate between employer branding and employee retention, but that much like the relationship between employer branding and employee retention, there is no research that clearly states organizational trust has a positive and significant influence in mediating the influence between these two variables.

## Methods

The subjects of this research are employees of one of the largest manufacturing companies in Indonesia, which is a company engaged in the automotive sector. This company is the only one in Indonesia that has the right to be the sole agent for the world's leading motorcycle brand. How many samples will be used in this study will be based on Hair et al. (2010) which uses more than 100 samples as the minimum number in the use of this study. Formula by Slovin et al. (1993) this is the minimum recommended size for this research survey.


$$\begin{array}{r} n=N/\left(1+\left(Nxe{}^{\text{2}}\right)\right)\\ n=23.953/\left(1+\left(23.953x0.05^{2}\right)\right)\\ n=23.953/60,8825\\ n=393,429968\\ n=394 \end{array}$$

Based on the formula above, from a total of 23,953 employees, the minimum sample size is 394 employees. Part-time workers and employees of manufacturing companies comprise the sample unit in this study. Considering the number of samples to be studied, which can be done randomly, the researchers used random sampling in their research at this company.

## Result

**Figure 1:**
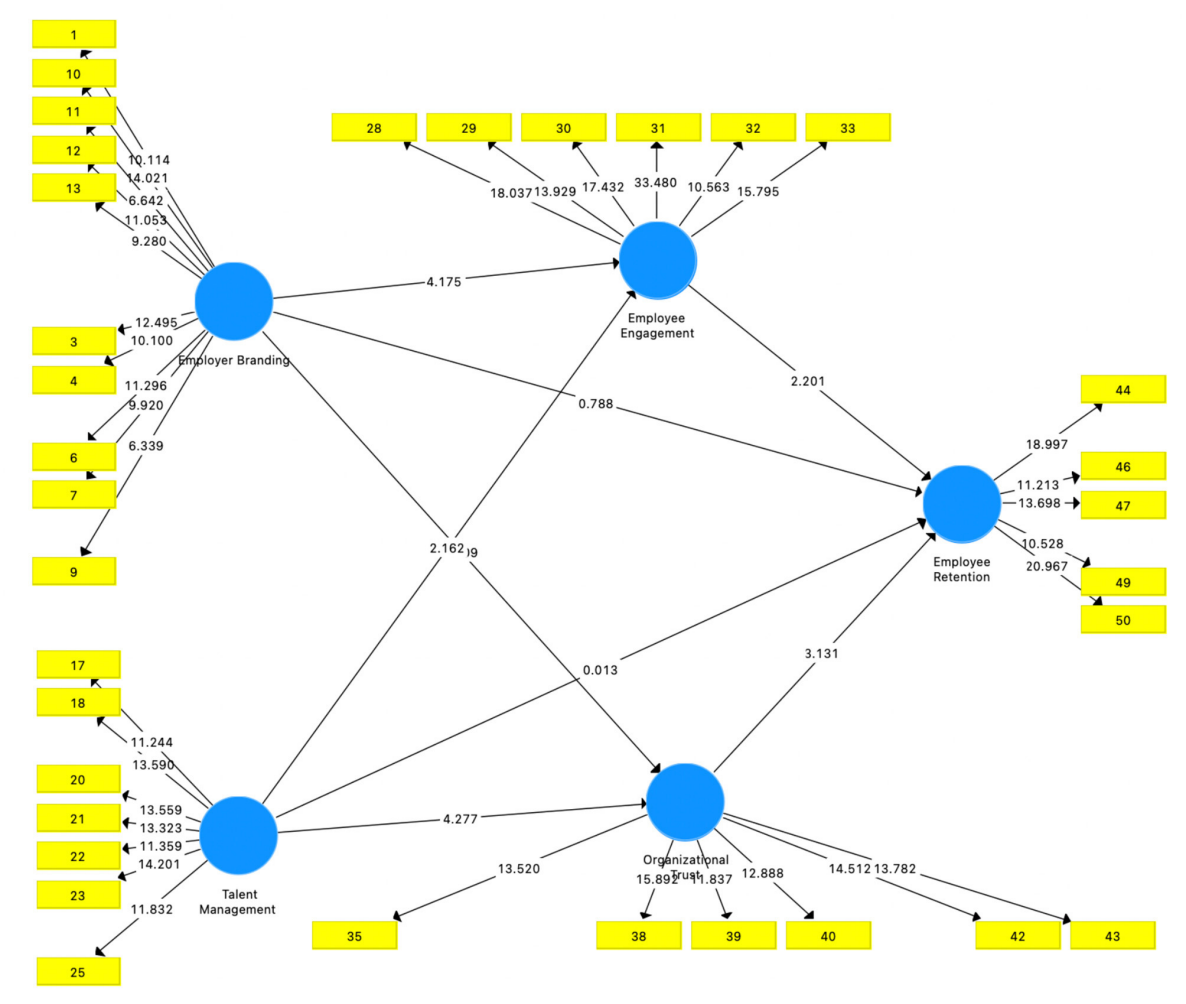
Bootstrapping.

At the data processing stage, validity, reliability, and discriminant validity tests have been carried out so that in the model there are several indicator items that are removed from the model because they do not meet the criteria that have been set to measure or explain the constructs in the model. Figure 1 shows the result of bootstrapping the final modified model.

The results of the bootstrapping above are used to test the hypothesis that all of the indicator items in all constructs have a statistical value >1.96. In conclusion, all of the indicator items above are able to measure the existing constructs. Meanwhile, to test the effect between variables, the statistical values from the Smart PLS analysis were compared with the table values. The following is a table that provides the results of the relationship between the constructs.

## Discussion

An important contribution to this research is the use of employee engagement and organizational trust as mediating variables based on consideration of the results of previous studies. This study found that employee engagement can positively mediate the influence of employer branding on employee retention. In contemporary research, which is characterized by intense business competition, engagement has received attention from both academics and practitioners and has been established as an important human resource intervention for the survival and growth of companies (Rai and Maheshwari, 2021). Many studies have been conducted to explore the various antecedents of engagement. Job-related antecedents are constructs, strategies, and conditions applied at the job or task level to develop engagement. It has been said in theories like the JCM (Job Characteristics Model) that work has a big impact on an employee's overall motivational potential by affecting their psychological states.

In this study, the contribution to organizational theory regarding organizational trust is an important element of social relations in an organization. At the interpersonal level, employees' trust in managers affects the results of their attitudes, behavior, and performance (Ozmen, 2019). Knowledge-based organizational trust among peers and commitment mediates the effect of employer branding on employee retention and also finds that trust in management partially mediates the relationship between employee-oriented HRM and employee behavior. Employer branding and employee retention are both important parts of this dissertation. One of the new things about adding organizational trust variables between these two things is that this model is used for companies in the automotive industry. From the psychological point of view of employee retention as a future factor for the company to retain talented employees in the future, human resource assets become a valuable asset but are often not visible in the eyes of management (Fahlevi, 2021). Understanding the psychology of employees to stay with the company is something that is rare in today's companies even though it is very much needed now.

## Conclusion

This research contributes as input to the automotive industry's business practices in increasing employee retention rates. The automotive industry, which has a high turnover rate, must innovate in the organization related to several main variables. The results obtained in this study are quite high, namely employee engagement and organizational trust. With the increase in these two variables, employer branding and talent management can be improved, which will have an impact on employees. Retention will increase as several variables in this research model will have a direct or indirect impact on the company's employee retention. The benefits of high employee retention for the company will reduce costs, be they recruitment costs or training costs. It is known that the automotive industry requires a large amount of training costs, especially on the part of production employees, which is a must for companies to provide training to their employees so that training costs can be reduced by increasing employee retention.

## Author Contributions

All authors listed have made a substantial, direct, and intellectual contribution to the work and approved it for publication.

## Conflict of Interest

The authors declare that the research was conducted in the absence of any commercial or financial relationships that could be construed as a potential conflict of interest.

## Publisher's Note

All claims expressed in this article are solely those of the authors and do not necessarily represent those of their affiliated organizations, or those of the publisher, the editors and the reviewers. Any product that may be evaluated in this article, or claim that may be made by its manufacturer, is not guaranteed or endorsed by the publisher.
